# Modeling Cervical Cancer Screening Strategies With Varying Levels of Human Papillomavirus Vaccination

**DOI:** 10.1001/jamanetworkopen.2021.15321

**Published:** 2021-06-30

**Authors:** David Robert Grimes, Edward M. A. Corry, Talía Malagón, Ciaran O’Riain, Eduardo L. Franco, Donal J. Brennan

**Affiliations:** 1 School of Physical Sciences, Dublin City University, Glasnevin, Dublin, Ireland; 2 Department of Oncology, University of Oxford, Oxford, United Kingdom; 3 Department of Gynaecological Oncology, Mater Misericordiae University Hospital, Dublin, Ireland; 4 Division of Cancer Epidemiology, Department of Oncology, McGill University, Montreal, Canada; 5 Department of Histopathology, St James’s Hospital, Dublin, Ireland; 6 Systems Biology Ireland, University College Dublin School of Medicine, Belfield, Dublin, Ireland

## Abstract

**Importance:**

Cervical cancer screening is a lifesaving intervention, with an array of approaches, including liquid-based cytology (LBC), molecular testing for human papillomavirus (HPV) infection, and combinations via parallel cotesting or sequential triage. Maximizing screening efficacy while minimizing overtreatment is vital, especially when considering how the HPV vaccine will affect the interpretation of results.

**Objectives:**

To estimate the likely outcomes of different screening modalities and to model how the increasing uptake of the HPV vaccine could affect the interpretation of screening results.

**Design, Setting, and Participants:**

This decision analytic model established a simple Markov model to compare the outcomes of different cervical cancer screening modalities on a simulated population of women (aged ≥25 years), considering different levels of HPV vaccination.

**Main Outcomes and Measures:**

The number of cases of cervical intraepithelial neoplasia (CIN) grade 2 and 3 detected and missed, the number of false positives, and the number of tests required to achieve a given level of accuracy. Positive and negative predictive values of different modalities were simulated under varying levels of HPV vaccination and therefore HPV prevalence.

**Results:**

In a simulated population of 1000 women aged 25 years and older with an HPV prevalence of 2%, HPV-based modalities outperformed LBC-based approaches, detecting 19% more true positives (HPV test sensitivity, 89.9% [95% CI, 88.6%-91.1%]; LBC test sensitivity, 75.5% [95% CI, 66.6%-82.7%]). While cotesting markedly reduced missed cases, detecting 29% more true positives than LBC alone (19.5 [95% CI, 19.3-19.7] per 1000 women screened vs 15.1 [95% CI, 13.3-16.5] per 1000 women screened), it unacceptably increased excess colposcopy referral by 94% (184.4 [95% CI, 181.8-188.0] false positives per 1000 women screened vs 95.1 [95% CI, 93.1-97.0] false positives per 1000 women screened). By contrast, triage testing with reflex screening substantially reduced false positives by a factor of approximately 10 (eg, HPV with LBC triage, 9.6 [95% CI, 9.3-10.0] per 1000 women screened). Over a lifetime of screening, reflex approaches with appropriate test intervals maximized therapeutic efficacy; as HPV vaccination rates increased, HPV-based screening approaches resulted in fewer unnecessary colposcopies than LBC approaches (HPV testing, 80% vaccine coverage: 44.1 [95% CI, 40-45.9] excess colposcopies; LBC testing, 80% vaccine coverage: 96.9 [95% CI, 96.8-97.0] excess colposcopies).

**Conclusions and Relevance:**

In this decision analytic model, the effectiveness of cervical cancer screening was dependent on the prevalence of cervical dysplasia and/or HPV infection or vaccination in a population as well as the sensitivity and specificity of various modalities. Although screening is lifesaving, overtesting or modalities inappropriate to the target population may cause significant harm, including overtreatment.

## Key Points

**Question:**

As human papillomavirus (HPV)–based cervical screening modalities change and HPV vaccination becomes more widespread, what are the likely outcomes for the interpretation of screening modalities?

**Findings:**

In this decision analytical model with a simulated population of women aged 25 years and older, HPV-based screening modalities detected more abnormal cervical cells than traditional liquid-based cytology (LBC) approaches, but they did so at the cost of increased false positives. However, as HPV vaccination increased, HPV-based modalities resulted in fewer unnecessary colposcopies than LBC methods.

**Meaning:**

These findings suggest that the ideal screening modality for a given population should account for HPV vaccination status to maximize the efficiency of screening.

## Teaser

This decision analytical model estimates the likely outcomes of different screening modalities and models the effect of growing uptake of the human papillomavirus (HPV) vaccine on the interpretation of screening results.

## Introduction

The impact of cervical cancer screening programs on conventional cytology has been dramatic. While primarily restricted to squamous cell carcinoma in women older than 25 years, the estimated 80% reduction in mortality associated with high-quality national screening programs illustrates its transformative effect.^1,2,3^ The evolution of cervical cancer screening to include human papillomavirus (HPV) testing is a desirable step^4^; HPV DNA testing as a screening tool has superior sensitivity in detecting cervical intraepithelial neoplasia (CIN) grade 2 and 3.^5^ Less emphasis has been placed on the high prevalence of HPV infection, and suboptimal implementation of primary HPV screening may result in increased referrals to colposcopy. As national programs transition to HPV testing, this necessitates reeducating the screened population regarding the benefits and limitations of cervical screening.^6^

False negatives can have detrimental consequences on women who receive cervical screening, and it is crucial to determine what represents acceptable false-negative rates within programs.^7,8^ This is further complicated by legal standards in the United Kingdom and Ireland requiring that screeners have “absolute confidence” in negative tests.^9,10^ However, screening tests are far from perfect,^11^ and while HPV testing is more sensitive than liquid-based cytology (LBC), the decreased specificity and potential for increased incidence of false-positive cases increases the potential for unnecessary and possibly deleterious interventions.

Considering the future of screening is crucial because HPV vaccination is already reducing the prevalence of CIN globally. While relatively recent, its effects have been dramatic; a recent study in a Scottish cohort found an 88% reduction in cervical disease due to vaccination,^12^ and modeling studies suggest Australia could virtually eliminate cervical cancer in coming decades due to its vaccination program.^13^ As prevalence decreases, the proportion of positive results that will be false positives will increase relative to the true positives, with emerging evidence of this in younger women.^12,14,15^ Implications of increased vaccination uptake must also be considered for any viable screening modality, as these shape the interpretation of results and clinical judgement.

Cervical screening is a lifesaving endeavor, but benefits and risks of different approaches must be carefully balanced to ensure maximum efficacy and to reduce the potential for overtreatment. As HPV vaccination becomes more widespread, this will affect the interpretation of results, and it is crucial we quantify this. Accordingly, we simulated outcomes of various screening modalities, modeling the proportion of CIN grade 2 and 3 cases detected and missed with different strategies. We also investigated the number of false-positive and false-negative results, quantifying likely excess referrals to colposcopy and risks of overtreatment. Finally, we investigated the association of HPV vaccination with the efficacy of current and future modalities and quantified how this could affect the interpretation of screening results.

## Methods

### Model Structure and Parameters

In this work, we simulated screening using a Markov mathematical model to estimate likely outcomes of different modalities and implementations. As the model is entirely feed forward, it is akin to a decision tree, but the Markov implementation with absorbing states is useful for quantifying final distributions with respect to transition probabilities, as outlined in the eAppendix in the Supplement. Outcomes were simulated for a hypothetical cohort of 1000 women with an assumed prevalence of CIN grade 2 or higher of 2%. This is a simplification, as natural history models show significant variation in the prevalence of CIN grade 2 or higher with age and nationality^16^; however, the 2% prevalence rate is broadly representative and appropriate to assess screening performance in a randomly selected cohort^11^ and can be modified for specific situations with the supplementary code provided. The full model description, schematics, transition probabilities, and parameter values are given in eFigure 1, eFigure 2, eFigure 3 in the Supplement. The 95% CIs were determined from confidence intervals for test sensitivities and specificities found in the literature, which were perpetuated through to the model results. As this study involved only simulated patient outcomes with no use of patient records or personal data, it was exempt from requiring ethical approval in accordance with Dublin City University’s research ethics committee guidelines. This study followed the Consolidated Health Economic Evaluation Reporting Standards (CHEERS) reporting guideline.

There are 3 especially relevant parameters to any screening test in our model: prevalence, sensitivity, and specificity (Table 1). These parameters determine the positive predictive value (PPV) and the negative predictive value (NPV) estimated by the model. The PPV is the probability that a positive test is a true positive, and the NPV is the probability that a negative test a true negative, defined in more detail in the eAppendix in the Supplement. As prevalence decreases, a positive result is increasingly likely to be a false positive, and PPV thus decreases. Conversely, as prevalence of a disease decreases, the NPV of testing tends to increase.

**Table 1.** Parameter Values for All Simulations

Parameter class	Parameter (symbol)	Value, %^a^
Prevalence, ie, proportion of the population with condition of interest	Prevalence of CIN grade ≥2 in typical population (P)	2
	Prevalence of detectable high-risk HPV in typical population (h)	8.4
	CIN grade ≥2 attributable to testable HR-HPV (v)	95.0
Sensitivity, ie, proportion of positive cases correctly identified as such	LBC test for CIN grade ≥2 (Sn_L_)	75.5 (95% CI, 66.6-82.7)
	HPV test for high-risk HPV (Sn_H_)	94.7
	HPV test for CIN grade ≥2(Sn_E_)	89.9 (95% CI, 88.6-91.1)
Specificity, ie, proportion of negative cases correctly identified as such	LBC test for CIN grade ≥2 (Sp_L_)	90.3 (95% CI, 90.1-90.5)
	HPV test for high-risk HPV (Sp_H_)	96.0 (95% CI, 95.7-96.1)
	HPV test for CIN grade ≥2 (Sp_E_)	89.9 (95% CI, 89.7-90.0)

Abbreviations: CIN, cervical intraepithelial neoplasia; HPV, human papillomavirus; LBC, liquid-based cytology.

^a^ All values taken from literature or inferred from literature values. See eAppendix in the Supplement for details of sources and ranges.

### Modeling Screening Outcomes

#### Primary Testing, Triage, and Cotesting

**Figure 1.**
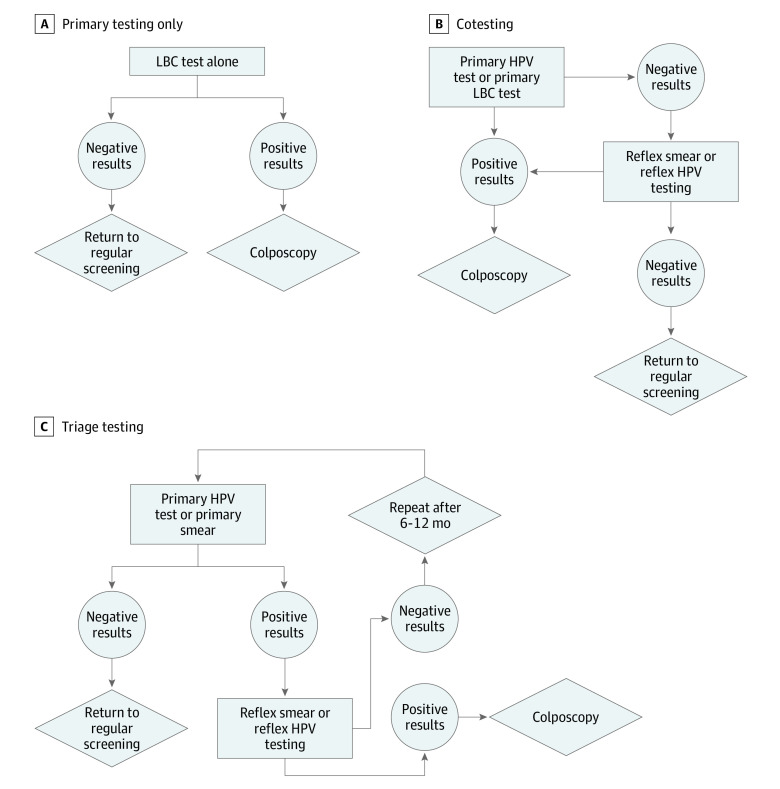
Flowcharts for Primary Testing Only, Cotesting, and Triage Testing These are general schemas, and there is large variation worldwide in exact implementation. HPV indicates human papillomavirus; LBC, liquid-based cytology.

Worldwide, there are numerous different approaches to cervical screening, differing even inside a country. Thus, we concentrated on general methods for illustration. The LBC test alone is illustrated in Figure 1A: a positive LBC test is referred to colposcopy. Conversely, primary LBC may be performed with reflex HPV test, where atypical squamous cells of undetermined significance (ASCUS)–positive results on a Papanicolaou test are then tested for HPV. For primary HPV testing, a positive result is followed by cytological examination; if abnormalities are then detected, referral is made to colposcopy. Another option with some clinical use is cotesting, in which both HPV and LBC tests are performed. There are different ways to perform cotesting, but 1 implementation is that a positive result in either test instigates either a scheduled retesting or elevation to colposcopy, illustrated in Figure 1B. Reflex triage approaches are illustrated in Figure 1C. In this case, an LBC screening can be performed and ASCUS results interrogated with a reflex HPV test, with positive results referred to colposcopy, or the converse can occur (HPV test with reflex LBC, as is the recommendation in Ireland). In addition to this, we also modeled the likely specific outcomes for 3 different regional and national screening programs; that of Ontario, Canada (LBC screening only), Ireland (HPV with LBC reflex), and the United States (cotesting). We assessed the following screening outcomes in the model: (1) lesions missed per 1000 women (false negatives); (2) theoretical maximum excess colposcopy referrals per 1000 women screened (false positives leading to unnecessary colposcopy referral); (3) PPV and NPV of a given implementation; and (4) total number of tests undertaken per 1000 women.

#### Repeated Testing of Negative Results

In principle, the risk of false negatives can be reduced by performing retests. As detailed in the eAppendix in the Supplement, repeated testing with LBC modalities constantly reduces the number of missed cases of CIN grade 2 and 3 cells, but it increases false positives. To our knowledge, such a method has not been previously implemented in precisely such a fashion, but it is worth noting that repeated testing of negative results is similar to asking women who screen negative to come back for another regular screening after a certain interval, similar to what is recommended in most countries. For example, in instances with a positive HPV result, women are tested again 6 to 18 months after this result. The retest can take several forms, ie, another HPV test (United Kingdom^17^ and Australia^18^), LBC (the Netherlands^19^), or both (United States^20^).

While the model here does not attempt to reproduce the natural history of HPV infection, it can be used to illuminate the clinical value and consequences of multiple screenings for a woman over time whether she has cervical disease or not. While absolute confidence in a negative result is impossible, it is possible to conclude with high certainty that a woman does not have CIN grade 2 or higher. We selected a confidence threshold (*t_s_*) of less than 0.1%, consistent with an NPV of greater than 99.99%, a threshold close to that of recent US guidelines.^21^ However, in each screening round, the prevalence (*p*) of CIN grade 2 and 3 in the cohort changes from *p_o_* to *p*(*n*), reducing missed cases but increasing false positives. The explicit formula for this is outlined in the eAppendix in the Supplement, and we simulated likely outcomes of cautionary retesting of negative results, ascertaining screening rounds required to achieve a threshold of *t_s_* and implications for colposcopy rates.

#### The HPV Vaccine and Screening Accuracy

Prophylactic HPV vaccination substantially reduces CIN prevalence. This has implications for both the PPV and NPV of screening tests. To model the association of vaccine uptake with screening performance, we took the pooled estimated reduction in HPV incidence from 29 modeling studies, as previously described in the literature.^22^ In these simulations, vaccine efficacy ranged from 90% to 100%, and this variation had little impact on pooled estimates.^22^ The upper and lower bands of these models were then used to estimate expected HPV prevalence under different levels of vaccine uptake (presuming vaccination against HPV subtypes 6, 11, 16, and 18), and then we calculated the PPV and NPV for different modalities.

### Statistical Analysis

All simulations were coded and performed with MATLAB version R2021a (Mathworks) and Mathematica version 12.1 (Wolfram Research) with standard packages. No statistical tests were performed.

## Results

### Comparison of Screening Modalities and Implementations

As shown in Table 2, triage testing reduced excess colposcopy referrals per 1000 women screened by approximately a factor of 10 compared with LBC or HPV testing alone (eg, LBC alone vs HPV with LBC triage, 95.1 [93.1-97.0] per 1000 women vs 9.6 [95% CI, 9.3-10.0] per 1000 women) and slightly increased the false negatives (eg, LBC alone vs HPV with LBC triage, 4.9 [95% CI, 3.5-6.7] per 1000 women vs 6.4 [95% CI, 5.1-8.0] per 1000 women). Comparing the sensitivities of LBC testing (75.5% [95% CI, 66.6%-82.7%) and HPV testing (89.9% [95% CI, 88.6%-91.1%]) suggests that HPV testing alone would detect 19% more cases than LBC (Table 1; eAppendix in the Supplement), with slightly more colposcopy referrals (98.9 [95% CI, 98.0-101.0] per 1000 women vs 95.1 [95% CI, 93.1-97.0] per 1000 women). Triage outcomes were the same regardless of the primary test, which may have economic consequences. For example, 1000 women receiving primary HPV tests requires 1000 HPV tests and 117 triage LBC tests. Conversely, when LBC is primary, 1000 LBC and 110 HPV tests are required. Cotesting increased true cases detected, detecting 29% more cases than LBC alone (19.5 [95% CI, 19.3-19.7] per 1000 women vs 15.1 [95% CI, 13.3-16.5] per 1000 women), but at the cost of increasing false-positive rate by 94% relative to LBC testing alone (184.4 [95% CI, 181.8-188.0] false positives per 1000 women vs 95.1 [95% CI, 93.1-97.0] false positives per 1000 women) and by almost 20-fold relative to triage approaches (9.6 [95% CI, 9.3-10] per 1000 women). The likely region-specific outcomes for the 3 different screening programs modeled were found to be broadly similar to the generic implementations detailed in Table 2 and are outlined in detail in eTable 1 to eTable 3 in the Supplement.

**Table 2.** CIN Grade 2 or 3 Detection Statistics for a Simulated Cohort of 1000 Women

Test type	PPV, % (95% CI)	NPV, % (95% CI)	False negatives per 1000 women, No. (95% CI)	False positives, ie, excess colposcopies, per 1000 women, No. (95% CI)
LBC test only	13.7 (12.1-15.1)	99.5 (99.3-99.6)	4.9 (3.5-6.7)	95.1 (93.1-97.0)
HPV test only^a^	15.4 (15.2-15.5)	99.8 (99.5-99.9)	2.0 (1.9-2.1)	98.9 (98.0-101.0)
HPV with LBC triage^b^	58.7 (55.3-60.1)	99.4 (99.2-99.5)	6.4 (5.1-8.0)	9.6 (9.4-9.8)
LBC with HPV triage^b^	58.7 (55.3-60.1)	99.4 (99.2-99.5)	6.4 (5.1-8.0)	9.6 (9.4-9.8)
Cotesting, HPV followed by LBC^c^	9.6 (9.5-9.7)	99.9 (99.6-99.9)	0.5 (0.3-0.7)	184.4 (181.8-188.0)
Cotesting, LBC followed by HPV^c^	9.6 (9.5-9.7)	99.9 (99.6-99.9)	0.5 (0.3-0.7)	184.4 (181.8-188.0)
**Screening rounds required to achieve *t_s_* <0.1% or NPV >99.99%**				
**Test type**	**Rounds required**	**Total tests required per 1000 women, No. (95% CI)**^**d**^	**False negatives per 1000 women, No. (95% CI)**	**False positives, ie excess colposcopies, per 1000 women, No. (95% CI)**
LBC test only	3	2690 (2688-2694)	0.3 (0.1-0.7)	258.4 (253.6-263.2)
HPV test only^a^	2	1883 (1881-1884)	1.0 (0.9-1.0)	137.7 (135.9-141.5)
Cotesting, HPV followed by LBC	1 (combined)	1883 (1881-1884)	0.5 (0.3-0.7)	184.4 (181.8-188.0)
Cotesting, LBC followed by HPV	1 (combined)	1890 (1890-1890)	0.5 (0.3-0.7)	184.4 (181.8-188.0)
Reflex testing, HPV followed by LBC	4	4274 (4267-4290)	1.0 (0.9-1.1)	34.3 (34.0-35.3)

Abbreviations: CIN, cervical intraepithelial neoplasia; HPV, human papillomavirus; LBC, liquid-based cytology; NPV, negative predictive value; PPV, positive predictive value; *t_s_*, confidence threshold.

^a^ HPV tests without reflex are not typically used but were shown for completeness.

^b^ Secondary test used as a triage test for initially positive results. These results are for the first round of screening plus triage and do not include subsequent management of triage negative women.

^c^ In cotesting simulation shown here, both tests were performed and a positive on either was referred to colposcopy. In more recent implementations, positive results can trigger a repeated cotest rather than immediate colposcopy, so this table shows worst-case scenario for excess colposcopy.

^d^ Total tests are the number of initial tests (1000) plus follow-up tests. For example, cotesting HPV followed by LBC requires 1000 initial HPV tests plus 883 (95% CI, 881-884) LBC tests.

### Implications of Test Modality and Frequency on Overscreening and Missed Positives

Table 2 also shows the number of test iterations required to reach an NPV of greater than 99.99% and indicates that while multiple screening rounds increased the detection rate, overdiagnosis also increased. For example, 3 rounds of LBC improved detection relative to a single round (19.7 [95% CI, 19.3-19.9] vs 15.1 [95% CI, 13.3-16.5]) but required a factor of 2.690 (95% CI, 2.688-2.694) more tests, with 172% the false-positive rate, translating to more than 150 extra colposcopies per 1000 women screened compared with a single screening (258.4 [95% CI, 253.6-263.2] false positives per 1000 women vs 95.1 [95% CI, 93.1-97.0] false positives per 1000 women), rendering it an unsustainable approach.

Test frequency is also an important consideration in determining the probability that a woman would receive an incorrect screening result. The cumulative probability of a false-positive result over a screening lifetime (assuming screening begins at age 25 years and ceases at 70 years) for a woman without CIN grade 2 or 3 and the probability of missing successive true, persistent CIN grade 2 or 3 for different modalities and intervals (ie, 1 year, 3 years, and 5 years) are given in the eAppendix in the Supplement. Triage testing resulted in fewer false positives but missed more cases of CIN grade 2 or 3. In contrast, cotesting reduced the number of missed cases of CIN grade 2 or 3 but at the cost of increasing the false-positive rate and number of referrals to colposcopy (eAppendix in the Supplement). These can be somewhat ameliorated by reducing screening frequency, as outlined in the eAppendix in the Supplement.

Triage tests themselves have some nuance that must be considered. Table 3 depicts the likely outcome of HPV primary testing with LBC reflex, considering the expedited retesting process that results from a positive HPV infection status. Outcomes and times to detection with triage are shown in Table 3, illustrating that retesting with both HPV and LBC detected more cases than an HPV retest alone and substantially more cases than a single LBC retest. Another important consideration for triage tests is the primary test; while outcomes were the same, testing order slightly affected the total number of tests undertaken (Table 3). Depending on the cost differential between HPV and LBC screening, this might be economically relevant.

**Table 3.** Possible Triage Outcomes With Expedited Retesting for a Woman With CIN Grade 2 or 3

Triage type	Probability, % (95% CI)	Outcome	Time to CIN grade 2 or 3 detection
Initial screening (HPV/LBC triage), HPV- and CIN grade 2 or 3–cytology positive	71.5 (63.1-78.4)	Colposcopy	Immediate
HPV detected, CIN grade 2 or 3–cytology negative	23.2 (16.4-31.6)	Expedited retest	Expedited test dependent
HPV missed, no cytology triage^a^	5.3 (NA)	False negative	Next screening cycle at earliest
HPV-only expedited retest; HPV detected^a^	94.7 (NA)	Colposcopy	6-18 mos after initial screening
Probability of missing CIN grade 2 or 3 after retest	1.2 (0.9-1.7)	NA	NA
LBC-only expedited retest; CIN grade 2 or 3 detected	75.5 (66.6-82.7)	Colposcopy	6-18 mos after initial screening
CIN grade 2 or 3 missed	24.5 (17.3-33.4)	False negative	Next screening cycle at earliest
Probability of missing CIN grade 2 or 3 after retest	5.7 (2.8-10.6)	NA	NA
HPV and LBC expedited retest; either HPV or CIN grade 2or 3 detected	98.7 (98.2-99.1)	Colposcopy	6-18 mos after initial
Both HPV and CIN grade 2 or 3 missed	1.3 (0.9-1.8)	False negative	Next screening cycle at earliest
Probability of missing CIN grade 2 or 3 after retest	0.3 (0.2-0.6)	NA	NA

Abbreviations: CIN, cervical intraepithelial neoplasia; HPV, human papillomavirus; LBC, liquid-based cytology; NA, not applicable.

^a^ Probabilities inferred from high-risk HPV test sensitivity value, as derived in eAppendix in the Supplement.

### Association of the HPV Vaccine With Screening Accuracy

**Figure 2.**
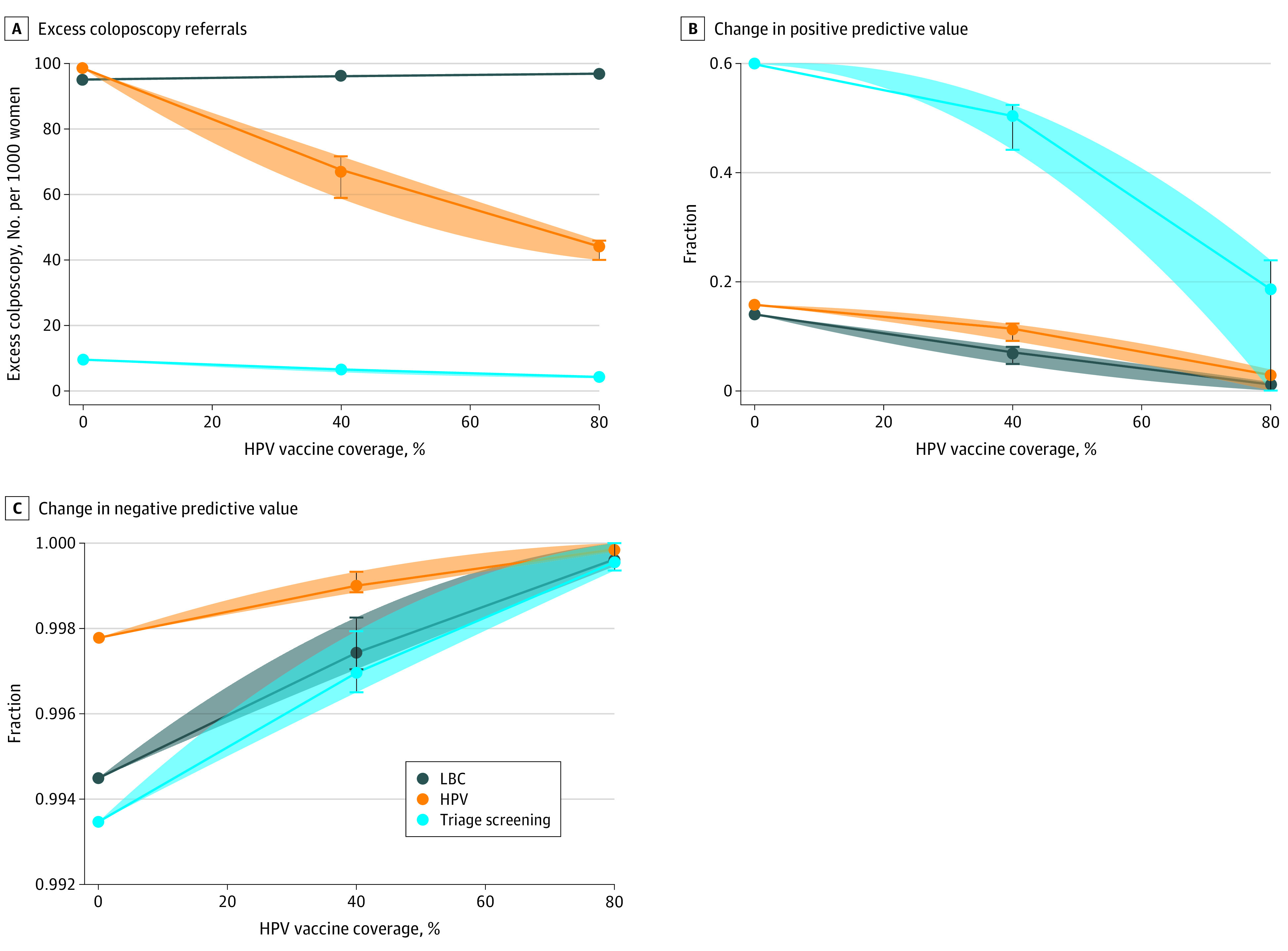
Association of Human Papillomavirus (HPV) Vaccine Uptake Rates With Excess Colposcopy Referrals, Positive Predictive Values, and Negative Predictive Values HPV-only screening is not typically performed but is included for completeness. The confidence envelopes in each panel refer to the range of estimates from 29 different models.^22^

Figure 2A depicts the association of screening with the number of false positives (ie, excess colposcopies) for HPV, LBC, and triage testing as vaccination rates increased. With LBC testing, excess colposcopies slightly increased as HPV incidence diminished (40% vaccine coverage, 96.1 [95% CI, 96.0-96.4] excess colposcopies; 80% vaccine coverage, 96.9 [95% CI, 96.8-97.0] excess colposcopies). By contrast, false positives from HPV and triage testing decreased as HPV incidence decreased (HPV testing: 40% vaccine coverage, 67.5 [95% CI 58.7-71.6] excess colposcopies; 80% vaccine coverage, 44.1 [95% CI, 40-45.9] excess colposcopies; triage testing: 40% vaccine coverage, 6.6 [95% CI, 5.7-6.9] excess colposcopies; 80% vaccine coverage, 4.3 [95% CI, 3.4-4.4] excess colposcopies). Figure 2B and Figure 2C show the PPV and NPV change with vaccination rates for these tests. Confidence envelopes were derived from a selection of natural history models, as previously described. As vaccination rates increased, the PPV of both tests decreased, while the NPV increased. HPV testing appeared to be the superior modality, as it resulted in fewer false positives than LBC testing. This could further be improved by implementing triage testing, and the results of this simulation suggest that HPV testing is a superior method of screening as vaccination rates increase and HPV infection decreases.

## Discussion

Cervical screening is a lifesaving intervention, but it must be applied judiciously to have maximum benefit while minimizing consequences of overtreatment in false-positive cases. In this study, we examined advantages and limitations of different screening methods. Decisions regarding which modality to use is not only a scientific question but one of appropriate allocation of limited public health resources.^23^ Primary HPV testing at a 3-year interval has been demonstrated to have, at a minimum, equivalence with cotesting every 5 years.^24^

Results of this analysis suggest that the false-positive rate of primary testing^25,26^ can be reduced with triage testing and that this utility increases as vaccination rates grow. Both triage modalities had only 10% the false-positive rate of LBC, detecting 90% of the cases LBC would detect. As these approaches yield essentially the same result, a screening program could implement them as their resources allowed. That triage outcomes are the same regardless of primary test has economic implications. While the total number of tests required differed by only a small amount, if there were substantial differential cost between modalities, then the optimum could be selected to minimize these without causing harm. If, for example, HPV tests were much more expensive than LBC tests, then taking a primary LBC approach for triage would be cost-saving. Alternatively, if cytology was a limiting resource, then an HPV-primary approach is more suitable.

The benefit of triage testing is the reduced number of excess colposcopies performed at a slightly reduced detection rate. One potential approach to increase detection is to perform surveillance and expedited retesting of women with negative triage results, which our model suggests would enable detection of the most prevalent cases of CIN grade 2 or 3. While cotesting resulted in improved detection ratio relative to LBC alone (a 29% improvement), it nearly doubled the false-positive rate (a 94% increase). This could prove excessively expensive and ultimately detrimental to public health because the increased rate of detection is associated with an amplified false-positive rate. While this modality reduced missed cases of CIN grade 2 and 3 cells, this analysis suggests it would not be viable, resulting in needless harm, as other authors have warned.^27^

This raises important ethical questions regarding the safety of any screening program. When an asymptomatic population is invited into a screening program, there remains an ethical obligation to maximize the probability that they exit the program with a reduced cancer risk and minimal harm.^28^ Increasing referrals to colposcopy is likely to lead to overtreatment of dysplastic lesions with associated consequences on fertility and obstetric outcomes, including a 2-fold increased risk of preterm birth.^29^ Overdiagnosis resulting from screening has long been recognized as a serious issue^16,30^ with screening programs, although it remains difficult to quantify.^31^ The results of this work should be useful in elucidating potential harms and benefits.

While the model presented here is useful for quantifying detection statistics, it is important to consider the limitations of this analysis. For the false-positive and false-negative lifetime probability, we did not model natural history, and there is an implicit assumption that test results are independent from previous test results. The model is not adapted to estimate the accuracy of screening at different ages or to assess the risk of progression or regression of CIN between screening opportunities. However, the results are likely a good approximation of the worst-case scenarios, ie, the risk of a woman with persistent CIN grade 2 or 3 receiving a negative result and the risk of a woman who remains negative receiving a positive result. This important assumption requires consideration, as it is plausible that there are simply some CIN grade 2 or 3 lesions that may never be detected with LBC or HPV testing because of characteristics of the lesion, such as low volume or low viral load. This affects the cumulative probability of a false negative and false positive.

A crucial point to acknowledge is that all screening modalities have inherent limitations—those that maximize detection are most likely to lead to false positives. Those reducing the incidences of false detection also reduce detection of CIN grade 2 or 3. Considering HPV triage with LBC screening, it appears that expedited retesting of HPV-positive results outside the regular screening cycle of 6 to 18 months helps to ameliorate the reduced detection ratio of triage tests while minimizing false positives and excess colposcopy referrals. This analysis also suggests that performing LBC-only retesting of triage results tends to detect less disease than HPV retesting or both HPV and LBC retesting.

In designing a screening program, one must be cognizant of the potential harms as much as benefits. The advent of HPV testing has had huge implications for cervical screening,^11,32^ which are quantified further in this article. The question of testing intervals was beyond the scope of this work, but it was briefly alluded to in the analysis of false-positive and false-negative cumulative probability illustrated for 1-year, 3-year, and 5-year intervals in the eAppendix in the Supplement. A recent study^33^ found that reducing the testing window interval does more harm than good, leading to overscreening with needless risk, excess costs, and overtreatment. Other authors^34^ have suggested that rescreening after a negative primary HPV test should occur no sooner than every 3 years, with Dillner et al^23^ reporting that intervals of even 6 years were safe and effective. The 2018 US guidelines for HPV screening^20^ recommend a minimum interval of 5 years between routine screening tests. As the present analysis illustrates, we would expect that extending this interval has only a small impact on the rate of missed cases of CIN grade 2 or 3 rate in most cases, while substantially reducing false positives.

As HPV testing becomes cheaper and more common, it is vital to consider how it is best implemented in screening. Evidence from recent multicenter studies^5,35^ indicates that HPV-based screening provides greater protection against invasive cervical carcinomas relative to LBC. Results in this work support the hypothesis that HPV screening every 5 years could reduce the number of unnecessary colposcopy and biopsy procedures compared with frequent LBC, cutting costs and reducing the number of invasive unnecessary procedures. There is also ample evidence that negative high-risk HPV tests provide greater and longer reassurance of low risk of abnormal results than negative cytology results,^34,36^ with authors suggesting that primary high-risk HPV screening can be considered as an alternative to current US cytology-based cervical cancer screening methods. Certainly, the results of this analysis support the contention that HPV testing can strongly increase the performance of cervical screening and, when correctly deployed, can also reduce potential harms of over-screening.

The staggering international success of the HPV vaccine is already apparent,^37^ and countries with high uptakes of the HPV vaccine are already seeing a decrease in rates of precancer and, most recently, abnormal cervical cells. A recent cohort study by Lei et al^38^ found that the PPV of cytology in Sweden was significantly reduced for girls who received the vaccination, with sharper decreases in PPV seen for girls vaccinated at younger ages. This emerging data agree with the theoretical predictions of this work, suggesting these findings have immediate practical applications. The falling prevalence of CIN grade 2 or 3 as HPV infection decreases due to vaccination has deep implications for how we interpret future tests; as this analysis indicates, the primary consequence of decreasing HPV infection rates is that across all modalities, positive results are less likely to be informative. This analysis also suggests that HPV testing is superior as infection rates decrease, resulting in fewer false positives than LBC testing. This is likely to be important in planning the future of screening programs. The model outlined in this work has applications here too, to help estimate the confidence that should be afforded a particular screening result under varying levels of population prevalence.

The requirement to provide accurate information on the outcomes of any alteration to screening programs can be illustrated by the psychosocial impact, which can cause additional stress and anxiety for those participating in screening.^39,40,41^ Unfortunately, the discrepancy between society’s expectation of screening programs and actual sensitivities exist, demonstrating the importance of public education.^42^ It is worth noting that physicians and health care professionals are also frequently underinformed about the benefits and limitations of screening programs,^43,44^ and confusion can easily arise. While screening is an extraordinary measure that saves lives, it is important to understand its fundamental limitations so that maximum benefit and minimum misunderstanding can be derived from any national program.

Cervical screening comes with inherent uncertainty, irrespective of the modality used. This work may help elucidate some optimal strategies for screening, but screening, while lifesaving, cannot be expected to be perfect. It is worth being clear that perfect detection is a mathematical impossibility; there is an inherent trade-off in strategies that increase detection, as they inevitably lead to a disproportionate rise in false positives, with needless overtreatment, as has been seen recently in the Netherlands.^45^ This is particularly relevant in the context of legal requirements in some jurisdictions, such as Ireland, where following legal action regarding disputed cervical cytology findings, the high court ruled that screeners must have “absolute confidence” in negative results,^10^ despite no evidence of wider systematic quality issues within laboratories participating in the screening program. Such a stipulation is impossible, and as this analysis shows, even striving to get close to this standard is likely to result in more harm than good. This is neither conducive to public health nor sustainable. It also has the potential to muddy public expectations and understanding of screening and what it can realistically achieve.

### Limitations

This study has limitations. Simulations assumed a uniform prevalence of CIN grade 2 or higher in the general population, which is a simplification, as natural history models show significant variation in prevalence with age and nationality.^16^ However, the prevalence value of 2% used in this work is generally representative and thus appropriate to assess screening performance in a randomly selected cohort.^11^ This limitation can also be overcome by varying prevalence parameters for specific groups of interest, with relevant equations for this given in the eAppendix in the Supplement.

## Conclusions

In this decision analytic model, the effectiveness of cervical cancer screening changed with the prevalence of population-level HPV vaccination as well as the effective sensitivity and specificity of the selected testing modality. Screening is a vital undertaking if we are to reduce cervical cancer mortality, and its strengths and limitations must be seen in context so that benefit can be maximized. This analysis should prove useful in optimizing approaches and demonstrating complexities of different implementations so that informed decisions can be made. Moreover, the balance of benefits and harms from screening will inevitably decrease in parallel with the decrease in cervical lesion prevalence, which will necessarily lead to rethinking what we accept today as test characteristics, age at start, age to exit, and frequency of screening.^46^

## Notes

Supplement. **eFigure 1. **Transition Probability for LBC Testing **eFigure 2. **Transition Probability for Cotesting **eFigure 3. **Transition Probability for Triage Testing **eAppendix.** Literature and Derived Parameter Estimation **eTable 1. **Simulated OCSP Screening Outcomes per 1000 Women **eTable 2. **Simulated US Screening Outcomes per 1000 Women Assuming Cotesting **eTable 3. **Simulated Cervicalcheck (Ireland) Screening Outcomes per 1000 Women eReferences.
