# Supplementary material for: Modeling Cervical Cancer Screening Strategies With Varying Levels of Human Papillomavirus Vaccination
Source: JAMA Netw Open. 2021 Jun 30;4(6):e2115321. doi: 10.1001/jamanetworkopen.2021.15321 (PMC8246311; doi:10.1001/jamanetworkopen.2021.15321)
Supplement: Supplement. — eFigure 1. Transition Probability for LBC Testing eFigure 2. Transition Probability for Cotesting eFigure 3. Transition Probability for Triage Testing eAppendix. Literature and Derived Parameter Estimation eTable 1. Simulated OCSP Screening Outcomes per 1000 Women eTable 2. Simulated US Screening Outcomes per 1000 Women Assuming Cotesting eTable 3. Simulated Cervicalcheck (Ireland) Screening Outcomes per 1000 Women eReferences. [file jamanetwopen-e2115321-s001.pdf]

Supplemental Online Content

Grimes DR, Corry EMA, Malagón T, O'Riain C, Franco EL, Brennan DJ. Modeling cervical cancer screening strategies with varying levels of human papillomavirus vaccination. *JAMA Netw Open.* 2021;4(6):e2115321. doi:10.1001/jamanetworkopen.2021.15321

eFigure 1. Transition Probability for LBC Testing

eFigure 2. Transition Probability for Cotesting

eFigure 3. Transition Probability for Triage Testing

eAppendix. Literature and Derived Parameter Estimation

eTable 1. Simulated OCSP Screening Outcomes per 1000 Women

eTable 2. Simulated US Screening Outcomes per 1000 Women Assuming Cotesting

eTable 3. Simulated Cervicalcheck (Ireland) Screening Outcomes per 1000 Women

eReferences.

This supplemental material has been provided by the authors to give readers additional information about their work.

eFigure 1. Transition Probability for LBC Testing

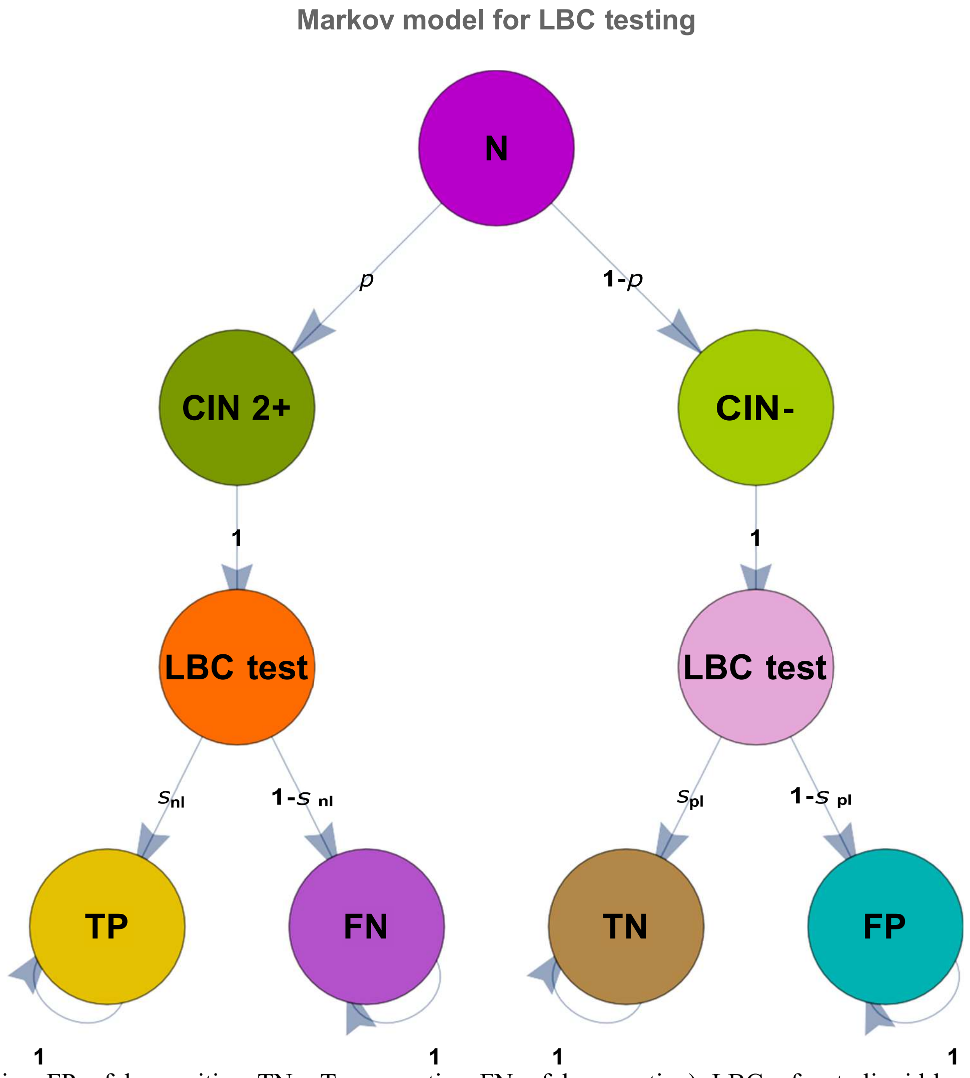

(TP = true positive, FP = false positive, TN = True negative, FN = false negative). LBC refers to liquid-based cytology, with other parameters specified in table 1.

© 2021 Grimes DR et al. *JAMA Network Open.*

eFigure 2. Transition Probability for Cotesting

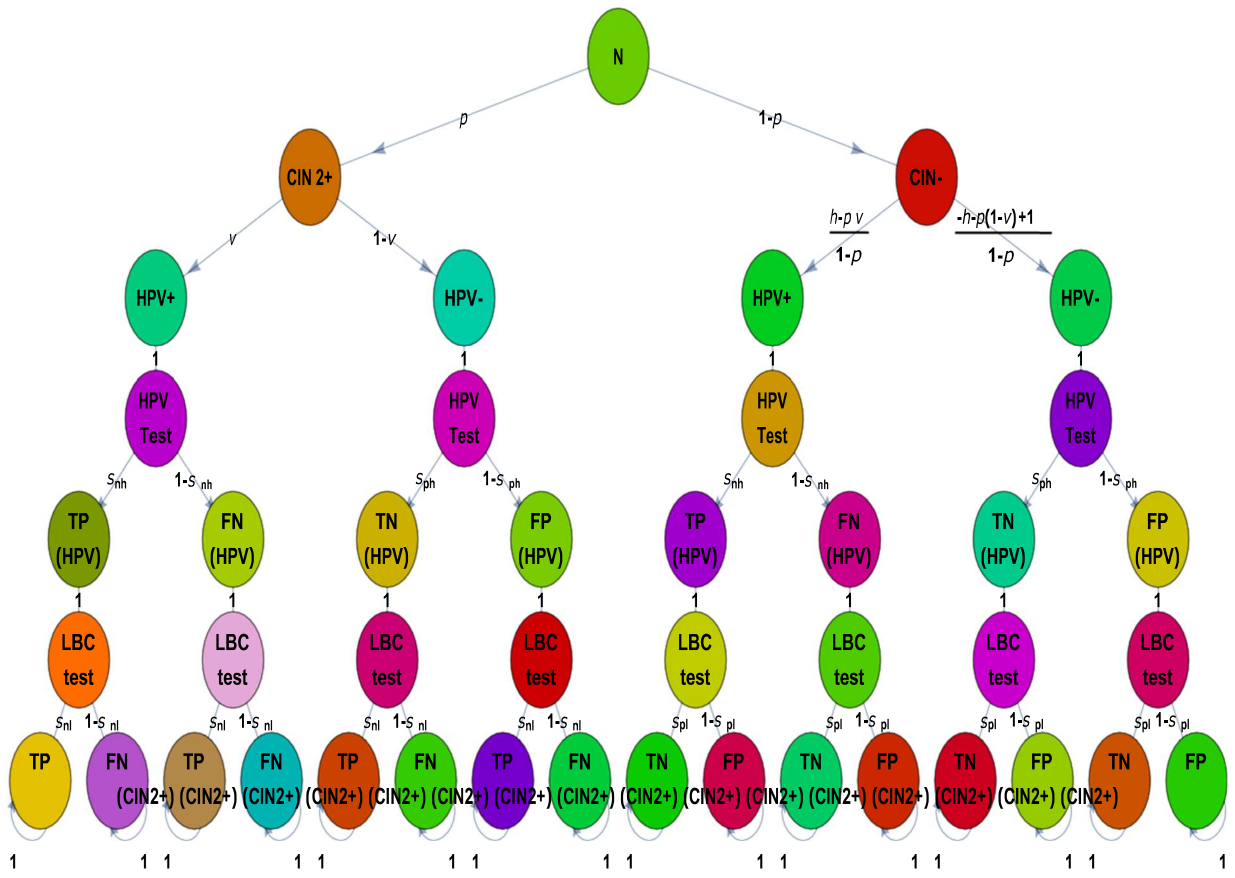

(TP = true positive, FP = false positive, TN = True negative, FN = false negative). LBC refers to liquid-based cytology, HPV to Human papillomavirus, with other parameters specified in table 1.

© 2021 Grimes DR et al. *JAMA Network Open.*

eFigure 3. Transition Probability for Triage Testing

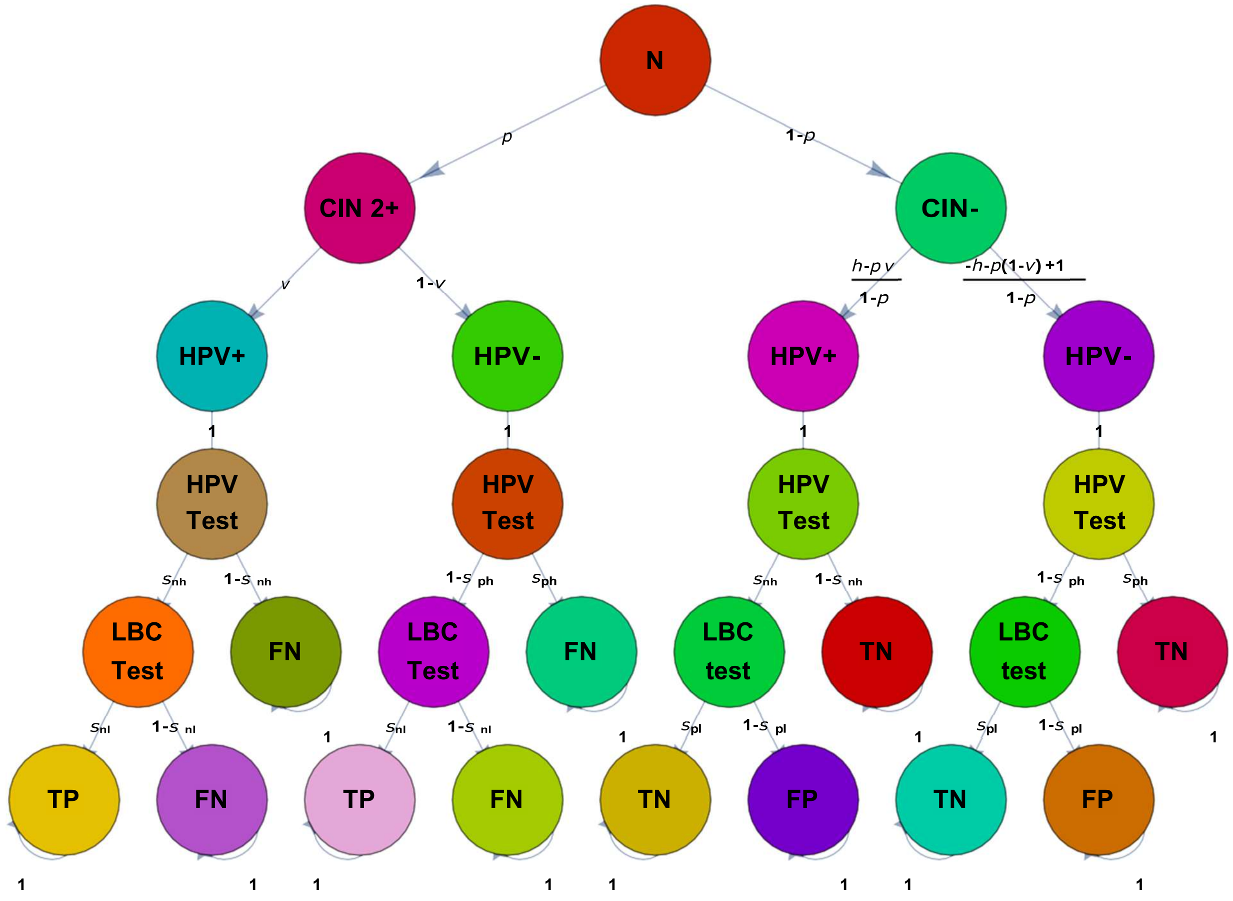

(HPV primary, TP = true positive, FP = false positive, TN = True negative,FN = false negative). LBC refers to liquid-based cytology, HPV to Human papillomavirus, with other parameters specified intable 1.

© 2021 Grimes DR et al. *JAMA Network Open.*

eAppendix. Literature and Derived Parameter Estimation

Table 1. Parameter values

Parameter	Symbol	Estimate with 95% CI where available
Prevalence of CIN2/3 in typical population[1]	p	2.0 (-)
Prevalence of detectable HR-HPV in typical population[a]	h	8.4 (-)
CIN2/3 attributable to testable hr-HPV	v	95.0 (-)
CIN2/3 Sensitivity (LBC)[1]	s_{nl}	75.5 (66.6 - 82.7)
CIN2/3 Specificity (LBC)[1]	s_{pl}	90.3 (90.1-90.5)
CIN2/3 Sensitivity (HPV testing)[1]	s_{ne}	89.9 (88.6-91.1)
CIN2/3 Specificity (HPV testing)[1]	s_{pe}	89.9 (89.7-90.0)
HR-HPV test Sensitivity[2]	s_{nh}	94.7 (-)
HR-HPV test Specificity[b]	s_{ph}	96.0 (95.7-96.1)

[a] Estimated value[2–5]. See below for details - [b] Derived value.

© 2021 Grimes DR et al. *JAMA Network Open.*

As depicted in table 1, most of the parameters required for this work could be taken directly from the literature. While the Cochrane report[1] and the investigations it reports upon provide an estimate for the sensitivity and specificity of HPV testing for CIN2/3 cases, it does not intrinsically yield the sensitivity and specificity of HPV testing to HPV infection, whichis a required parameter for this analysis. From literature[3–5], we estimate the proportion of the population with a detectable HPV infection to be about 0.08. We further note that a recent analysis[2] estimated that half of CIN2/3 HPV- results would have been recorded as CIN2/3, HPV+ if a more sensitive test had been employed. Factoring this into Cochrane data[1], this is equivalent to detecting an extra 1 of the 2 CIN2/3 cases missed. This yields a estimate for the proportion of CIN2/3 cases dueto detectable HPV types as (19/20) or $\nu = 0.95$, and yields a sensitivity estimate for detectable HPV with a 14 targeted type test as

$$s_n = 18/19 = 0.947 \quad (1)$$

and accordingly, a true detectable HPV prevalence rate in the population as

$$h = \frac{0.08}{s_n} = 0.084. \quad (2)$$

Finally, we can then estimate the specificity of the HPV test. The number of excess colposcopy, E, is given by

$$E = Nh \quad 1 - \frac{p_o \nu}{h} \quad s_n + N \quad 1 - h \quad 1 - \frac{(1-\nu)p_o}{1-h} \quad 1 - s_p \quad . \quad (3)$$

This can be readily re-arranged and solved to obtain s_p for HPV testing, as given in the main paper table 1.

False positives and false negatives with screening frequency

Under the assumption of independence of screening tests, we apply Poisson statistics and find the cumulative probability of at least one false positive after n tests is

$$f_p(n) = 1 - \exp(-n(1 - s_p)) \quad (4)$$

where s_p is specificity of the test. An equally important consideration is the probability of missing disease in successive tests. For a woman with non-regressing CIN2/3, the probability it will be missed with each test is the complement of the sensitivity (s_n) raised to the power of n, explicitly stated as

$$f_n(n) = (1 - s_n)^n . \quad (5)$$

As frequency is an important consideration in determining the lifetimes probability that one gets an incorrect screening result, we can model cumulative probability of a false positive result over a screening lifetime (assuming screening begins at 25 and ceases at 70) for a CIN2/3 negative woman and the probability of missing successive true, persistent CIN 2/3 for different modalities and intervals (1 year, 3 year, and 5 year). This is shown in figure 4.

Mathematical analysis of Triage testing

It is straightforward if rather tedious to establish formula for the outcome of triage tests. For N patients, we define s_{n_H} is the sensitivity of HPV testing to HPV infection and s_{p_H} the sensitivity. We further define s_{n_L} as LBC sensitivity to CIN2/3 and s_{p_L} as LBC CIN2/3 specificity. It can be shown that regardless of order of triage, the total of CIN2/3 missed, ω, and the excess colposcopy (false positives, ε) are the same for both approaches, given respectively by

$$\omega = Np_o \quad \nu \quad 1 - s_{n_h} s_{n_L} \quad + \quad 1 - \nu \quad 1 + s_{n_L} \quad s_{pH} - 1 \quad (6)$$

$$\varepsilon = N \quad 1 - s_{p_L} \quad h - p\nu \quad s_{n_H} + \quad 1 - h - (1 - \nu)p \quad 1 - s_{p_H} \quad . \quad (7)$$

© 2021 Grimes DR et al. *JAMA Network Open.*

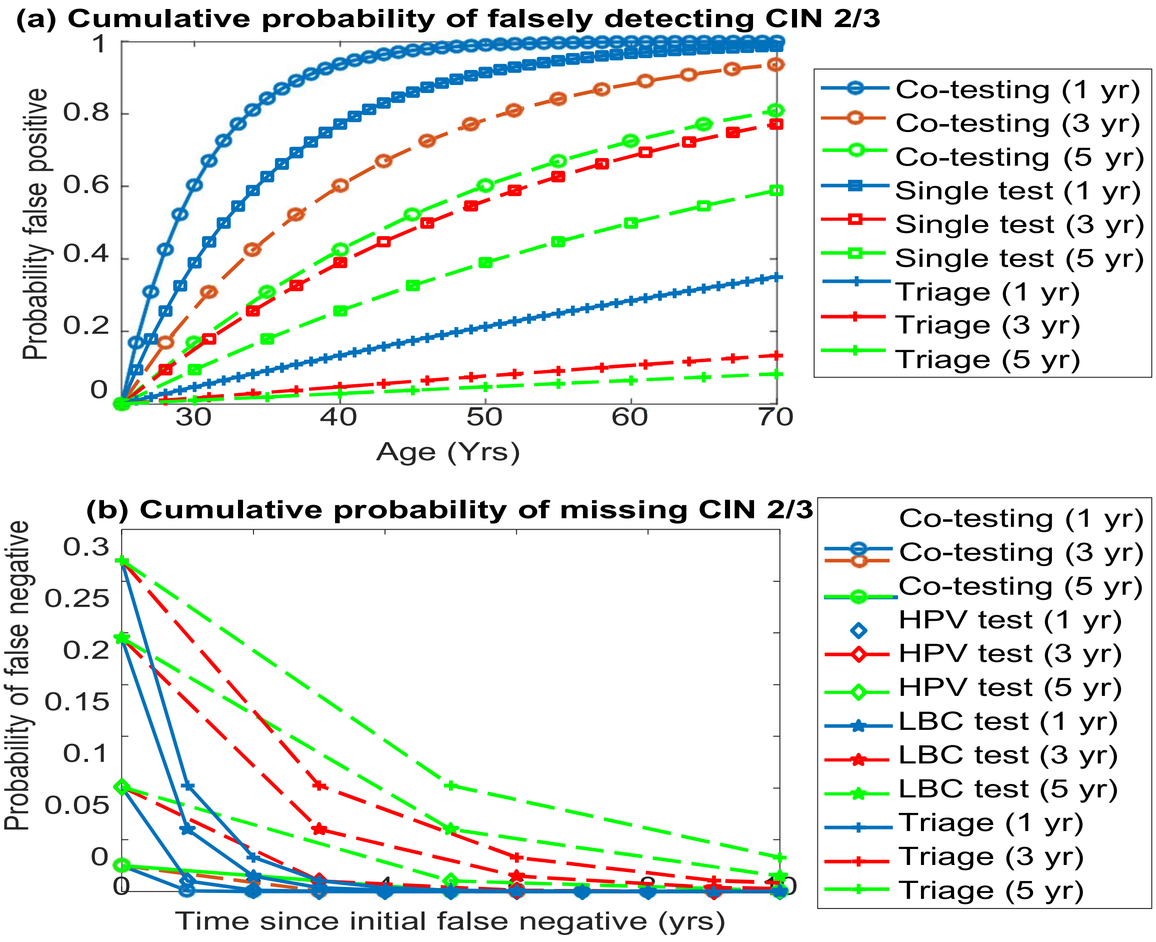

Figure 4. Cumulative probability of getting a false positive result with different modalities and screening intervals over a patient life-time, assuming screening begins at 25 and ends at 70. Note that results for single LBC / HPV tests are so similar they have been collapsed into one category (single test) for clarity, with caveat that HPV-only screening is not typically performed and shown only for completeness. (b) Cumulative probability of missing persistent CIN 2/3 for different modalities with different screening intervals, with the horizontal axis depicting number of years since the initial false negative result. Note that HPV only screening is shown for comparison despite limited clinical use.

In terms of excess colposcopy and positives missed, order (HPV primary with LBC reflex or LBC primary with HPV reflex) does not matter. But there is some variation with respect to the total number of tests performed. For HPV primary and LBC primary respectively, total tests performed are

$$t_{hp} = N \quad 1 + h s_{n_H} + \quad 1 - h \quad 1 - s_{p_H} \tag{8}$$

$$t_{lp} = N \quad 1 + p_o s_{n_L} + \quad 1 - p_o \quad 1 - s_{p_L} \quad . \tag{9}$$

Ideally, we wish to select modality so that total tests is minimised. This is highly dependent on prevalence of CIN2/3, itself a function of HPV infection rate. In the case of HPV primary testing, we can state

© 2021 Grimes DR et al. *JAMA Network Open.*

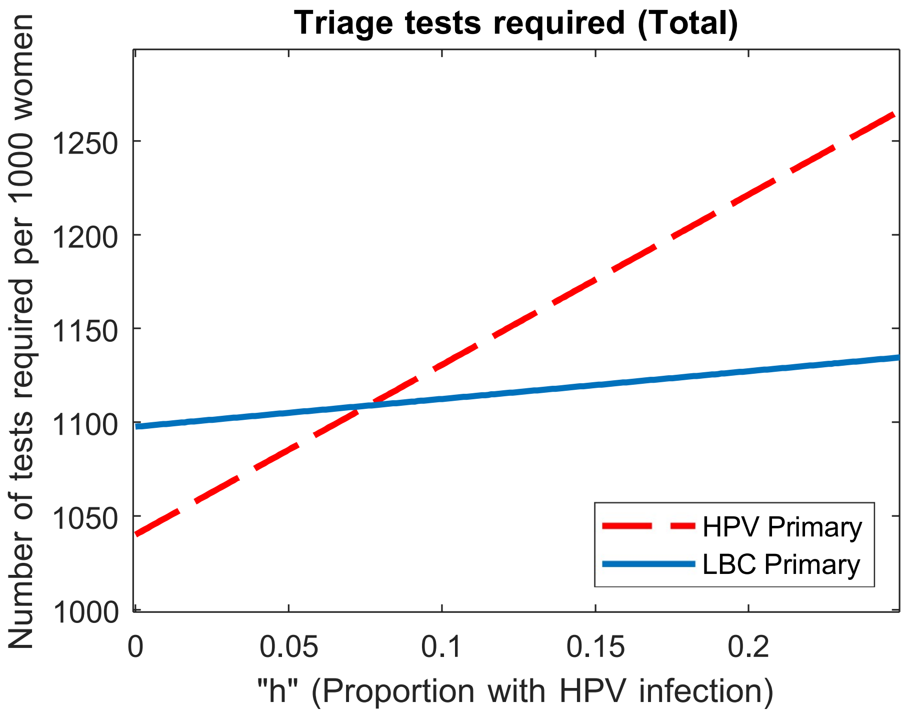

Figure 5. Impact of HPV infection rate on total number of triage tests required

$$\frac{\partial t_{hp}}{\partial h} = N\left(s_{n_H} + s_{p_H} - 1\right) \qquad (10)$$

and as the right-hand side of the identity is a positive constant, we can thus state that the number of tests required is a growing linear function of h, and define this constant C_1 for brevity. We assume that CIN2/3 infection is a linear function of HPV infection rate, so that $p = \varphi h + \lambda$. We can substitute this into t_{lp}, and write

$$\frac{\partial t_{lp}}{\partial h} = N\varphi\left(s_{n_L} + s_{p_L} - 1\right) \qquad (11)$$

and again, the right-hand side is a positive constant, designated C_2. From the prior section, we know that in a typical population, about 1 in 1000 CIN2/3 cases is not due to detectable HPV strains, allowing us to get $\lambda = 0.001$. Thus we can infer as previously discussed that $\varphi \approx 0.225$. It also follows that $C_1 >> C_2$, which means that the number of tests required grows more rapidly with HPV infection rate for HPV primary testing than primary LBC testing. One can show that below a threshold h infection rate h_t, primary HPV testing will result in fewer tests required. Above this threshold, $t_{hp} > t_{lp}$. This threshold is given by

$$h_t = \frac{s_{p_L} - s_{p_H} + \lambda\left(s_{n_L} + s_{p_L} - 1\right)}{s_{n_H} + s_{p_H} - 1 - \varphi\left(s_{n_L} + s_{p_L} - 1\right)} \qquad (12)$$

and for the parameters here, one can estimate $h_t = 7.60\%$ ($7.29 - 7.63\%$ to 95% confidence interval). This is actually lower a threshold than the h estimated in the paper, but as can be seen from supplementary figure 1, the number of tests is relatively similar in both cases for typical h.

Mathematical analysis of Co-testing

Co-testing can be modelled as the process of primary testing with one modality (LBC/HPV), followed by a single reflex test on

negative results with the complementary modality (LBC for HPV primary, HPV for LBC primary). It can be readily shown thatregardless of order of testing, the number of cases detected (d), number of excess colposcopies / false positives (ε), and numberof cases missed (ω) are given by

$$d = Np \quad v \; s_{n_H} + s_{n_L} - s_{n_L} s_{n_H} \quad + (1 - v) \;\; 1 - s_{p_H} + s_{p_H} s_{n_L} \tag{13}$$

$$\varepsilon = N \quad h - pv \quad 1 - s_{p_L} + s_{p_L} s_{n_H} \quad + \quad 1 - h - p + pv \quad 1 - s_{p_L} s_{p_H} \tag{14}$$

$$\omega = Np \quad v \;\; 1 - s_{n_H} \quad + (1 - v) \;\; s_{p_H} \qquad 1 - s_{n_L} \quad . \tag{15}$$

The total number of tests, however, will differ very slightly, depending on test order. For LBC and HPV tests initially performed, the total number of tests are given by

$$t_L = N \quad 1 + p \;\; 1 - s_{n_L} \quad + (1 - p) s_{p_L} \tag{16}$$

$$t_H = N \quad 1 + h \;\; 1 - s_{n_H} \quad + (1 - h) s_{p_H} \quad . \tag{17}$$

As with Triage testing, this will vary with prevalence and HPV infection rate. Using the same function ($p = \varphi h + \lambda$) and [illegible]

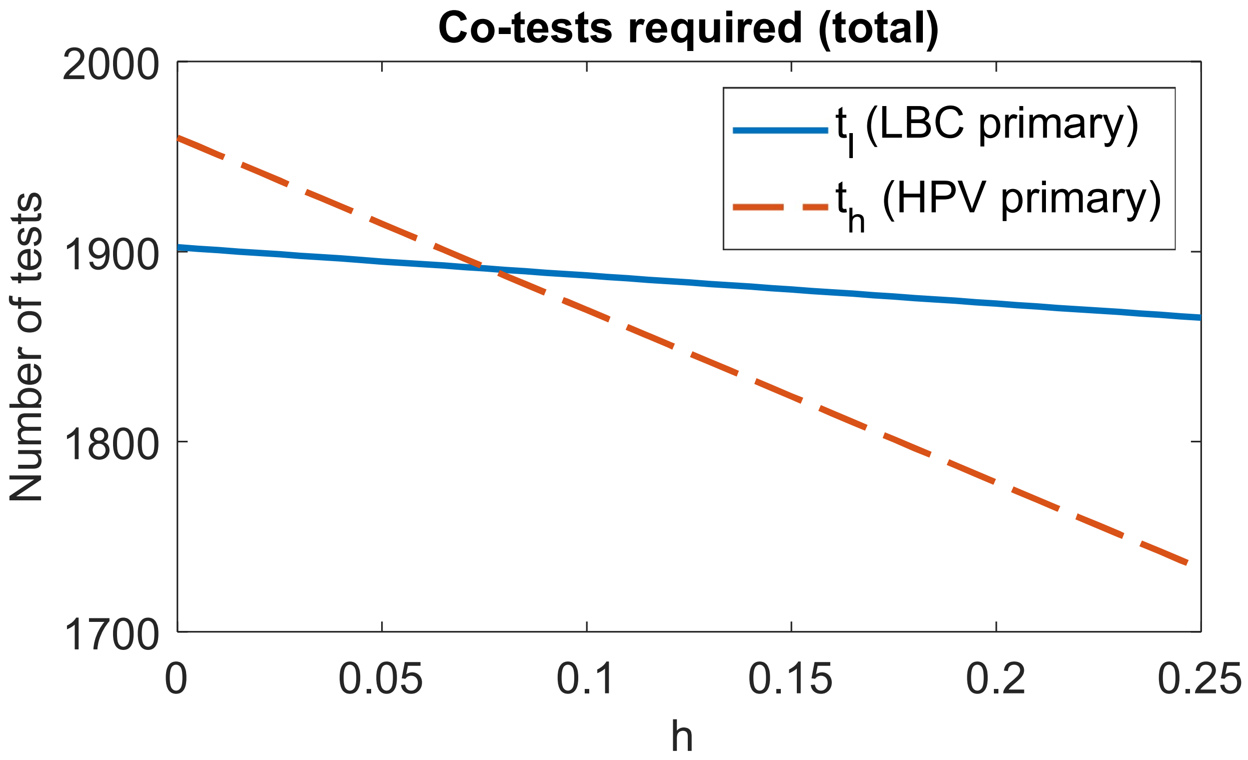

Figure 6. Impact of HPV infection rate on total number of co-tests required

Mathematical analysis of repeat negative testing

Repeat LBC testing

For repeated LBC testing, the prevalence of CIN2/3 changes upon each iteration. It can readily shown through analysis of recurrence relationships be shown that the prevalence at iteration n is given by

© 2021 Grimes DR et al. *JAMA Network Open.*

$$p(n) = \frac{p_o}{\left(\frac{s_p}{1-s_n}\right)^n (1 - p_o) + p_o} \tag{18}$$

where p_o is initial prevalence, s_n is test sensitivity and s_p test specificity. The final consideration is the number of extra tests required if a retest mandate was initialised. For an initial population to be screened of N_o, we define a function $g(n) = s_p + p(n)(1 - s_p - s_n)$, we can find the number of retests $r(n)$ at each iteration and the total number of tests after n iterations, $t(n)$, respectively by

$$r(n) = N_o \prod_{k=1}^{n-1} g(k) \tag{19}$$

$$t(n) = \sum_{k=1}^{n} r(k) \tag{20}$$

The total extra precancers detected after n tests, $\varphi(n)$ and total false positives after n tests, $\varepsilon(n)$ are given respectively by

$$\varphi(n) = s_n \sum_{k=1}^{n} t(k)p(k) \tag{21}$$

$$\varepsilon(n) = (1 - s_p) \sum_{k=1}^{n} t(k)(1 - p(k)) \tag{22}$$

Repeat HPV testing

Repeat HPV tests can be quantified neatly with the following relationship. For n tests, the number of missed positives ($\omega(n)$), false positives (excess colposcopy) ($\varepsilon(n)$) and total tests required per iteration, $t(n)$, are given by

$$\omega(n) = Np\left(v\left(1 - s_{nH}\right)^n + (1 - v)s_{pH}^n\right) \tag{23}$$

$$\varepsilon(n) = N\left((h - pv)\left(1 - s_{nH}\right)^{n-1} + (1 - h - p + pv)\, s_{pH}^{n-1} s_{nH}\right) \tag{24}$$

$$t(n) = N\left(h\left(1 - s_{nH}\right)^n + (1 - h)\, s_{pH}^n\right) \tag{25}$$

© 2021 Grimes DR et al. *JAMA Network Open.*

Regional / Country comparison

We can also simulate the outcome of different modalities employed by existent regions and countries, in a similar manner to that shown in table 2 of the main paper. Three different regimes are outlined below, and exact transition probabilities were taken from the Markov chains outlined at the end of this document, with parameters as given in main paper and supplementary material.

LBC testing - Ontario, Canada

The current Ontario Cervical Screening Program (OCSP) screening recommendations outline an LBC testing regime, similar to that described in main paper figure 1. Specifically, any high grade abnormalities are recommended for immediate colposcopy in women greater than 25 years of age[6]. Likely outcomes are shown in table 2.

Co-testing - USA

The USA: Practice in the USA varies markedly across the country, and co-testing regimes have in recent years been common in many jurisdictions. To see likely outcomes from recommended guidelines, we can look to the 2012[7] guidelines to see likely outcomes of screening with co-testing. Results of this analysis are shown in table 3. However, these results should be interpreted with the caveat that these algorithms are no longer recommended in the USA, whose screening protocols are now completely different from any other country.

Triage testing - Ireland

Ireland has a national screening programme, and has moved to HPV primary screening with LBC reflex. This triage schema recommends testing at 3 year intervals for women 25-30, and at 5 year intervals for those aged over 30[8]. Likely outcomes are shown in table 4.

eTable 1. Simulated OCSP Screening Outcomes per 1000 Women. (95% confidence intervals in brackets)

Screening result	True prevalence	Correctly identified	Missed	False detection	Outcome
Below CIN2	980	885 (883-897)	95 (93-97)	5 (3-7)	LBC test 3 years
CIN2+	20	15 (13-17)	5 (3-7)	95 (93-97)	Colposcopy

eTable 2. Simulated US Screening Outcomes per 1000 Women Assuming Cotesting (95% confidence intervals in brackets)

Screening result	True prevalence	Correctly identified	Missed	False detection	Outcome
LBC-, HPV-	915	792 (788-795)	122 (119-125)	3 (3-3)	Co-test at 3 years
LBC-, HPV+	65	56 (56-56)	9 (9-9)	38 (36-41)	Either repeat co-test in one year or colposcopy
LBC+, HPV-	1	1 (1-1)	0 (0-0)	86 (85-88)	Either repeat co-test in one year or colposcopy
LBC+, HPV+	19	14 (12-15)	5 (4-7)	9 (9-9)	Colposcopy

eTable 3. Simulated Cervicalcheck (Ireland) Screening Outcomes per 1000 Women (95% confidence intervals in brackets)

Screening result	True prevalence	Correctly identified	Missed	False detection	Outcome
HPV+ (Primary)	84	80 (80-80)	4 (4-4)	37 (36-39)	Reflex LBC
HPV+, LBC-	65	59 (59-60)	6 (6-6)	41 (39-45)	Repeat triage test after 12 months
HPV+, LBC+	19	14 (12-15)	5 (3-7)	8 (8-9)	Colposcopy
HPV-, LBC+	1	0 (0-0)	1 (1-1)	-	Missed CIN2+ - return to regular screening (3 years 25-30, 5 years 30+)
HPV-	916	879 (877-879)	37 (36-39)	3 (3-4)	Return to regular screening (3 years 25-30, 5 years 30+)

© 2021 Grimes DR et al. *JAMA Network Open.*

eReferences

1. Koliopoulos, G. *et al.* Cytology versus hpv testing for cervical cancer screening in the general population. *Cochrane Database Syst. Rev.* (2017).
2. Petry, K. U. *et al.* Evaluating hpv-negative cin2+ in the athena trial. *Int. journal cancer* **138**, 2932–2939 (2016).
3. Guan, P. *et al.* Human papillomavirus types in 115,789 hpv-positive women: a meta-analysis from cervical infection to cancer. *Int. journal cancer* **131**, 2349–2359 (2012).
4. Castle, P. E. *et al.* Performance of carcinogenic human papillomavirus (hpv) testing and hpv16 or hpv18 genotyping for cervical cancer screening of women aged 25 years and older: a subanalysis of the athena study. *The lancet oncology* **12**, 880–890 (2011).
5. Wright, T. C. *et al.* Primary cervical cancer screening with human papillomavirus: end of study results from the athena study using hpv as the first-line screening test. *Gynecol. oncology* **136**, 189–197 (2015).
6. Ontario cervical screening program (ocsp) screening recommendations summary (2020).
7. Massad, L. S. *et al.* 2012 updated consensus guidelines for the management of abnormal cervical cancer screening tests and cancer precursors. *J. Low. Genit. Tract Dis.* **17**, S1–S27 (2013).
8. Programme, N. S. S. Hpv primary screening algorithm (2020).
